# Bidirectional Mendelian randomization analysis of the genetic association between primary lung cancer and colorectal cancer

**DOI:** 10.1186/s12967-023-04612-7

**Published:** 2023-10-15

**Authors:** Zhihan Xiao, Zichen Wang, Tongyu Zhang, Yi Liu, Mingxuan Si

**Affiliations:** 1 Department of Cardiothoracic Surgery, Wuhu Second People’s Hospital, Wuhu, China; 2 Department of Thoracic Surgery, The First Affiliated Hospital of Nanjing Medical University, Nanjing, China; 3 Department of Digestive System, Anqing Municipal Hospital, Anqing, China; 4 Department of Thoracic Surgery, Renji Hospital, Shanghai Jiao Tong University School of Medicine, Shanghai, China

**Keywords:** Lung cancer, Colorectal cancer, Mendelian randomization, GRS

## Abstract

**Background:**

With the development and popularization of low-dose chest CT technology, the diagnosis and survival rates of patients with early lung cancer (LC) have significantly improved. The occurrence of colorectal cancer (CRC) as the second primary cancer (SPC) in primary lung cancer (PLC) survivors has become an essential factor affecting the prognosis of early LC. This study explored the potential association between PLC and CRC genetically, laying a foundation for developing SPC-CRC prevention strategies after primary early LC.

**Methods:**

Based on a two-sample bidirectional Mendelian randomization (MR) design, this study systematically screened genetic instrumental variables (IVs) based on the genome-wide association studies (GWAS) of PLC and CRC, applied inverse variance weighted (IVW) as the main method to assess the incidence association between the two cancers, and used a variety of other MR methods for supplementary analysis. Finally, the Genetic Risk Scores (GRS) method was used for secondary analysis to verify the results robustness further.

**Results:**

From LC to CRC forward MR analysis, 20 genetic IVs of overall LC, 15 genetic IVs of squamous cell lung carcinoma (LUSC), and 10 genetic IVs of adenocarcinoma of the lung (LUAD) were screened. In the reverse MR analysis from CRC to LC, 47 genetic IVs for overall CRC, 37 for colon cancer, and 25 for rectal cancer were screened. The IVW method and a variety of MR methods all found that overall LC and CRC were significantly associated at the genetic level. Subgroup analysis also showed that LUSC was associated with CRC. And the results of the GRS method were consistent with those of the main analysis, confirming the robustness of the study.

**Summary:**

Our MR study found an association between LC and CRC, with an increased risk of SPC-CRC following PLC, especially LUSC. Our study provides an essential basis for the precise prevention of SPC-CRC after PLC, suggesting that we should pay more attention to the population with a history of PLC in clinical work, and pay close attention to the incidence of SPC-CRC, and carry out intervention and treatment as soon as possible.

**Supplementary Information:**

The online version contains supplementary material available at 10.1186/s12967-023-04612-7.

## Introduction

Lung cancer (LC) is a highly prevalent malignancy and is the foremost cause of worldwide cancer-related mortality [1]. Non-small-cell lung cancer (NSCLC) is the predominant histological subtype of LC, accounting for 76% of LC. It encompasses a diverse range of cancer types, with the largest subgroups being adenocarcinoma of lung (LUAD) and squamous cell lung carcinoma (LUSC) [2]. With the advancement and widespread utilization of low-dose chest CT, the diagnostic rate for primary LC (PLC) has significantly increased, leading to a substantial number of patients being diagnosed with early-stage LC. Statistics from the Japanese Joint Committee of LC Registry Database indicate that in 2010, 18,973 patients received treatment for PLC in Japan. Among them, stage I patients accounted for 78.9% of the total [3, 4]. The study suggests that early-stage LC will become the predominant population for LC management with the widespread implementation of low-dose chest CT screening in high-risk groups.

Currently, surgery is the recommended treatment for patients diagnosed with stage I-IIIA NSCLC [5]. Lobectomy is considered the standard surgical approach and has been associated with a 5 year overall survival rate of 77–92% for clinical stage IA, 68% for IB, 60% for IIA, 53% for IIB, and 36% for IIIA [6]. In recent years, the Japanese Society of Clinical Oncology has conducted a series of prospective clinical studies on surgical treatment strategies for early-stage LC, with the most influential study, JCOG0802, exploring stage IA LC patients with a solid component greater than 50% and less than 2 cm in diameter. The findings indicate that segmental resection and lobectomy have comparable efficacy, as evidenced by a 5 year survival rate exceeding 90% (94.3% for segmental resection *vs* 91.1% for lobectomy). Further analysis of the causes of death revealed that second primary cancer (SPC) is the second leading cause of mortality after LC itself. It is also one of the main factors contributing to better 5 year survival rates for patients undergoing segmental resection than lobectomy, with colorectal cancer (CRC) being the most common type among all SPCs [7]. In addition, the National Cancer Institute conducted a multicenter intergroup trial for NSCLC, revealing that approximately 15% of stage I patients develop SPCs. Of particular concern in post-operative early-stage NSCLC patients is CRC, which ranks as the second most lethal SPCs [8]. The studies above indicate that as the early diagnosis and treatment system for PLC gradually improves, patients can attain long-term survival following surgery. However, the occurrence of SPCs poses a significant threat to postoperative patient survival. Observational studies suggest that CRC is one of the main types of SPCs after PLC surgery. However, due to the inherent limitations of observational studies, such as confounding factors, whether there is an association between PLC and the development of CRC at the genetic level remains to be seen [9].

Mendelian randomization (MR) is a widely utilized method of etiological inference in genetic epidemiology [10]. In recent years, with the further exploration of MR research methods, they have increasingly become an ideal approach for gene-level studies to infer pathogenic associations between two complex diseases. For example, 2021 Li et al. explored the association between rheumatoid arthritis and Parkinson's disease based on a genome-wide association study (GWAS) with a large sample, using MR analysis of two samples [11]. In the same year, Zhu et al. used MR to investigate the association between polycystic ovary syndrome and breast cancer and found that poly-cystic ovary syndrome was strongly associated with the development of triple-negative breast cancer [12].

In this study, we aim to utilize GWAS data of PLC and CRC to elucidate the correlation between these two cancers at the genetic level through a bidirectional two-sample MR analysis. Our study will provide a foundation for developing prevention strategies for CRC after early-stage PLC surgery in clinical practice.

## Materials and methods

**Fig. 1:**
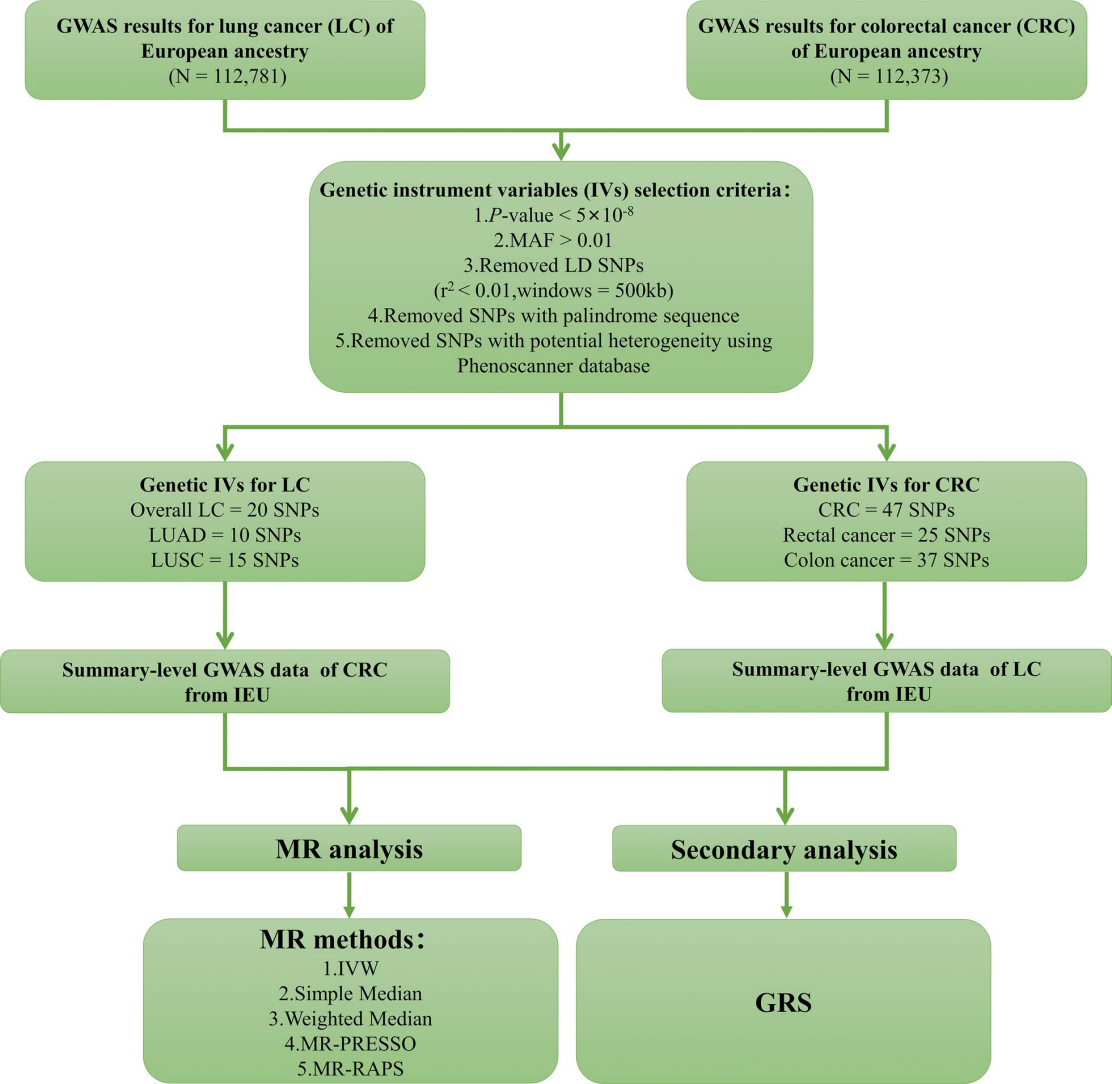
Study design and overview of our Mendelian randomization (MR) study. *LC* lung cancer, *CRC* colorectal cancer, *MAF* minor allele frequency, *IVW* inverse-variance weighted, *MR-PRESSO* Mendelian Randomization Pleiotropy RESidual Sum and Outlier, *MR-RAPS* Mendelian Randomization robust adjusted profile score, *GRS* Genetic risk scores, *LUAD* adenocarcinoma of lung, *LUSC* squamous cell lung carcinoma

The overview of the study design of the MR is displayed in Fig. 1. We estimated the cause effects of LC and CRC using inverse variance weighted (IVW), which was used as the primary method of analysis in this study. And we used genetic risk score (GRS) to validate the main results. Also, we applied various sensitivity analysis methods of two-sample MR to validate analysis results, including simple median, weighted median, MR-robust adjusted profile score (MR-RAPS), and MR-pleiotropy residual sum and outlier (MR-PRESSO).

### Sources of data

The genetic instrumental variables (IVs) for LC were derived from the largest sample size to date of the PLC GWAS published by James D. McKay*,* which used 14,803 cases and 12,262 controls of European descent to genotype on the OncoArray and combined the results with the previously published results from aggregated GWAS analysis of LC on 29,266 patients and 56,450 controls [13]. Regarding the reverse analysis, we obtained CRC-risk genetic IVs from two recent meta-analyses of GWASs on CRC risk [14]. The GWAS summary statistics of LC and CRC were downloaded from its public website “open GWAS” (https://gwas.mrcieu.ac.uk/). We used only freely accessible summarized data in this study; therefore, this study did not require ethical approval.

### Selection of IVs

The MR analysis evaluates the effect of a predictor on an outcome. There are three assumptions for a valid IVs—it must be: (a) associated with the exposure (the “relevance” assumption); (b) independent of the outcome given the exposure (the “exclusion restriction”); and (c) independent of all (both observed and unobserved) confounders (the “exchangeability” assumption) [15, 16]. If an IV is associated with a confounder of exposure and outcome, then there is a conflict with these assumptions, which may lead to potential biases and erroneous conclusions. Therefore, genetic IVs for overall LC, LUSC, LUAD, overall CRC, colon cancer and rectal cancer were constructed according to the following criteria [17]: (a) r^2^ measure of linkage disequilibrium (LD) among IVs < 0.01 at a 500 kb window (Genetic variants in close genomic locations tend to co-inherit, a phenomenon known as LD, when LD exists among genetic variants, the information provided by each genetic variant is not independent, and when these genetic variants are not independent of each other as IVs, the effect estimation will be biased); (b) *P* value less than the genome-wide significant level identified in the corresponding study (5 × 10^−8^, in the GWAS study, the criteria indicated an association between SNPs and disease); (c) minor allele frequency (MAF) > 0.01 (mutations are present in more than 1% of the population); (d) nonpalindromic single-nucleotide polymorphisms (SNPs, palindromic sequences are those in which SNPs in the forward and reverse strands of DNA have the same order of bases, in opposite directions. When the frequency of the outcome effect gene is low, it is not possible to infer whether the chain is in the forward or reverse chain); (e) removal of IVs associated with confounding factors using the PhenoScanner (in the MR analysis, IVs is likely to be associated with the outcome through confounding factors, and if the association between IVs and confounding factors is not excluded, the research results will be affected) [18].

### MR analyses

The principal analyses were conducted using the inverse variance weighted (IVW) approach. The IVW method, the most commonly used and mainstream method for MR analysis, use meta-analysis approach to combine ratio estimates of SNPs in an inverse variance weighted way and obtain an estimate of the effect of risk factors on outcomes [19, 20]. Ratio estimates are the ratio of the effect of a single SNP on the outcome divided by the effect on the risk factor (with all associations assumed to be log-linear) [21]. The IVW method provides reliable estimates when all IVs are valid, meeting the three core MR assumptions as provided above. IVW methods include the fixed-effects IVW and the random-effects IVW. If heterogeneity exists in the MR analysis, we will apply the random-effects IVW, which is not prone to weaker bias SNP-exposure association [22]. Additionally, the weighted median, simple median, MR-PRESSO, MR-RAPS and MR-Egger are used to assess whether LC and CRC are associated at the genetic level, and *P* < 0.05 is considered statistically significant. Weighted median and simple median method, which have the high tolerance for pleiotropic genetic variation that can obtain relatively stable effect values even when nearly half of the IVs are invalid. The key distinction between the two methods lies in their management of estimated medians, with the simple median method assigning equal weight to all values and the weighted median method incorporating weight for each value [22, 23]. MR-PRESSO method, which assumes that at least 50% of the genetic variants are valid genetic IVs, holding horizontal pleiotropy and the InSIDE assumption. In addition to identifying outlier genetic IVs, MR-PRESSO method can also provide adjusted estimation after removal outlier genetic variants [24]. In conclusion, the MR-PRESSO approach has the following three primary purposes [23, 25]: (1) “MR-PRESSO global test” to identify the extent of horizontal pleiotropy; (2) “MR-PRESSO outlier test” to exclude aberrant genetic variants (outliers) and estimate the corrected results; (3) “MR-PRESSO distortion test” to assess whether the discrepancy exists between the pre-corrected and corrected outcomes. The MR-RAPS with a Huber loss function can model the random-effects distribution of pleiotropic effects. Taking into account both systematic and idiosyncratic pleiotropy, the MR-RAPS method showed outstanding performance in numerical patterns. It is highly recommended as a practical tool for regular MR analysis, especially when dealing with complex traits that involve exposure and outcome [26]. MR-Egger regression method, which provides a weighted linear regression of the outcome coefficients on the exposure coefficients and can detect some violations of the standard instrumental variable assumptions and provide a non-violation-prone effect estimate [27].

### Genetic risk scores (GRSs)

To validate the above MR results, we conducted a secondary analysis by applying the GRS method. We conducted the analyses utilizing R (version 3.5.3) with the “gtx” R package (version 0.0.8 for Windows), whose grs.summary module has the GRS function. The grs.summary module merely used single SNP association summarized data obtained from the results of the GWAS analysis, which is similar to a method which regresses an outcome onto an additive GRS [25, 28]. For uncorrelated SNPs, the causal estimate α value can be estimated by $\alpha \approx \frac{\sum\upomega \beta {se}_{\beta }^{-2}}{{\sum }^{{\upomega }^{2}}{se}_{\beta }^{-2}}$, and the standard error se_α_ can be estimated by ${se}_{\alpha }\approx \frac{1}{{\sum }^{{\omega }^{2}}{se}_{\beta }^{-2}}$. Here, ω denotes the estimated effects on the intermediate trait or biomarker, and *β* values are estimated effects on the response variable or outcome with standard errors se_*β*_ [28].

### Horizontal pleiotropy and heterogeneity test

MR-Egger regression and the Cochran’s Q test were applied to estimate pleiotropy and heterogeneity, respectively. We eliminated the possibility that the MR-Egger intercept had a *P* value of less than 0.05 with the exclusion of possible horizontal pleiotropy. If the *P* value of Cochran’s Q test was less than 0.05, the final results of MR referred to a multiplicative random-effects model of IVW. Leave-one-out sensitivity analysis was also performed to further assess each IV’s independent potency. We considered a *P* value of less than 0.05 to indicate a statistically significant genetic association between exposures and outcomes. The strength of the association between SNP and the exposures are evaluated using the F statistic [29]. No weak IVs is present if the F statistic is > 10 (Additional file 3: Table S2).

A two-sided statistical analysis was conducted, and statistical significance was determined at *P* < 0.05. R version 4.1.2 and the packages “MendelianRandomization”, “TwosampleMR”, “RAPS”, “PRESSO” and “gtx” were used for all analyses [30].

## Results

### MR analysis results of LC to CRC

#### Screen and validation of IVs

In LC to CRC MR analysis, 4002 overall LC, 1176 LUAD, and 2789 LUSC IVs in the GWAS study reached significant differences (*P* < 5 × 10^–8^). The overall LC, LUAD, and LUSC IVs datasets identified 3912, 1122, and 2755 IVs that were nonpalindromic sequences, respectively (90, 54, and 34 palindromic sequences identified in the overall LC, LUAD, and LUAD IVs datasets, respectively). Based on the LD status between genetic variant loci, 25, 13, and 15 independent IVs associated with overall LC, LUAD and LUSC were selected without LD correlation (3887 overall LC, 1135 LUAD, and 2770 LUSC IVs are not LD independent. r^2^ < 0.01, window = 500 kb). Removal of IVs associated with confounders using the PhenoScanner database (smoking: rs3999544, rs55781567, rs56113850; alcohol consumption: rs17391694; BMI: rs71658797) [31, 32]. Ultimately, we identified 20 genetic IVs for overall LC, 10 for LUAD and 15 for LUSC (Additional file 3: Table S2).

#### MR results of overall LC to CRC

**Fig. 2:**
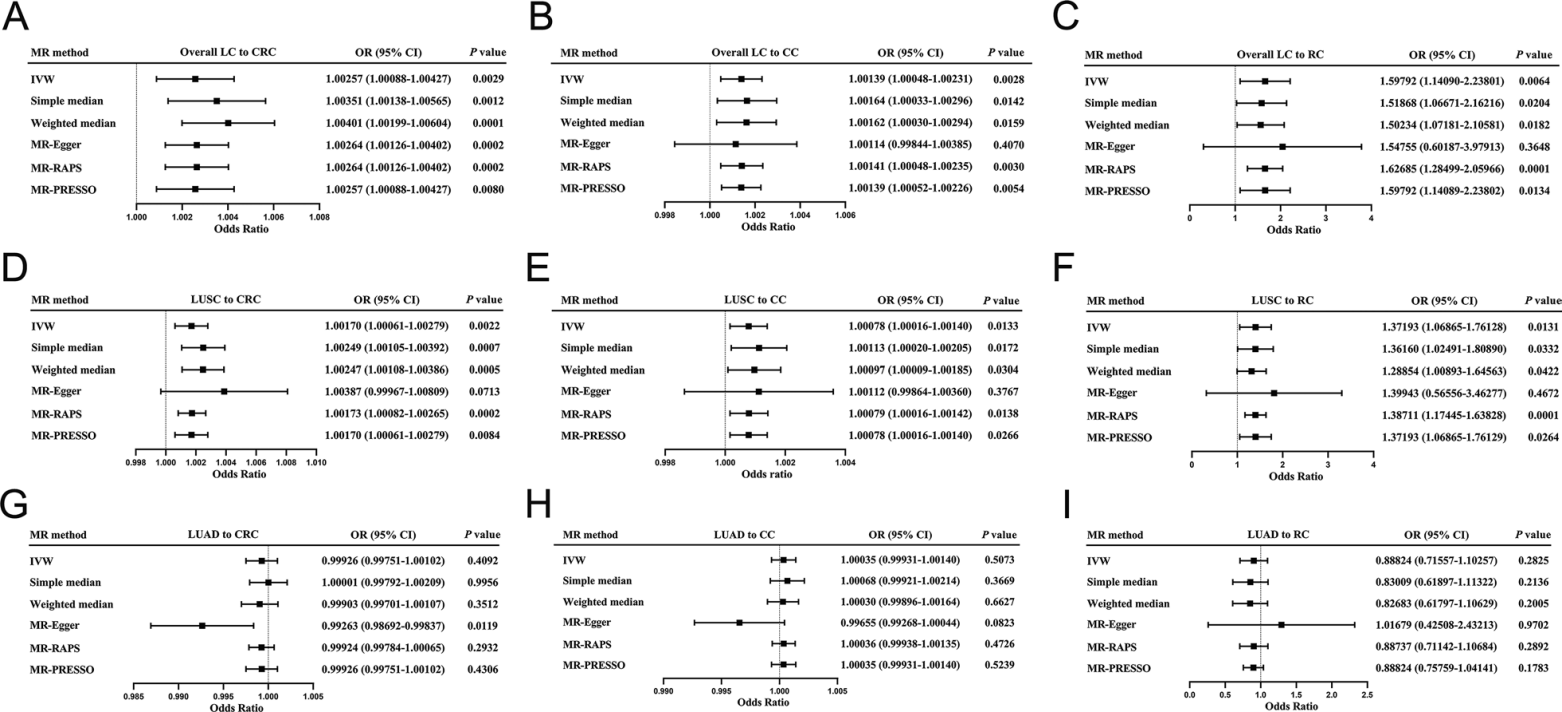
Forest plot of Two-Sample Mendelian Randomization study based on the MR method form LC to CRC. **A**, **B**, **C** Mendelian randomization estimates of genetically predicted overall LC on CRC (**A**), CC (**B**) and RC (**C**) risk. **D**, **E**, **F** Mendelian randomization estimates of genetically predicted LUSC on CRC (**D**), CC (**E**) and RC (**F**) risk. **G**, **H**, **I** Mendelian randomization estimates of genetically predicted LUAD on CRC (**G**), CC (**H**) and RC (**I**) risk. *LC* lung cancer, *CRC* colorectal cancer, *IVW* inverse variance weighted, *MR-PRESSO* Mendelian Randomization Pleiotropy RESidual Sum and Outlier, *MR-RAPS* Mendelian Randomization robust adjusted profile score, *LUAD* adenocarcinoma of lung, *LUSC* squamous cell lung carcinoma, *CC* colon cancer, *RC* rectal cancer

**Fig. 3:**
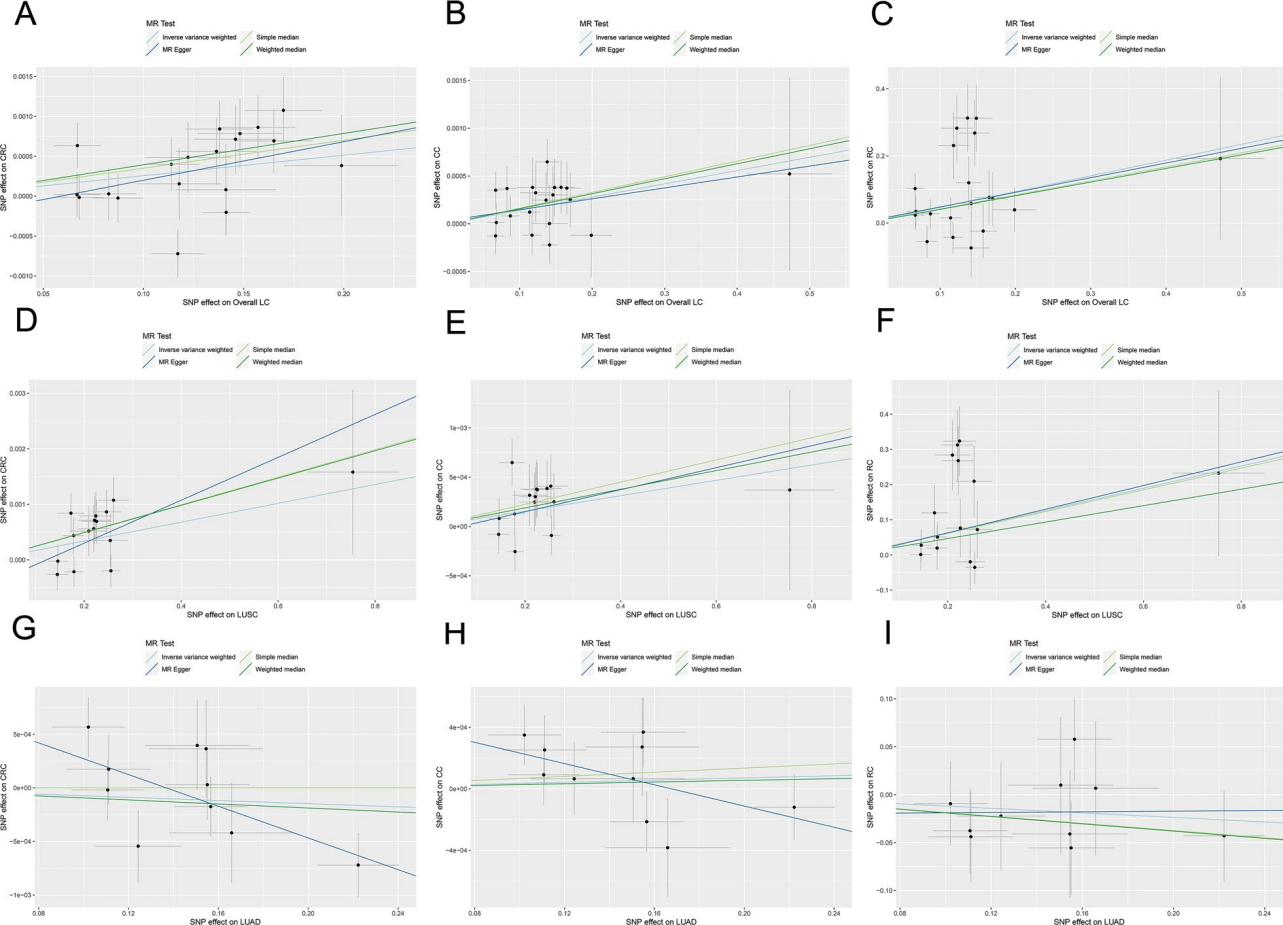
The scatterplots represent genetic IVs association between LC and CRC (Forward MR analysis). **A**, **B**, **C** Plots of the effect size of each single nucleotide polymorphism (SNP) of overall LC on CRC (**A**), CC (**B**) and RC (**C**) risk. **D**, **E**, **F** Plots of the effect size of each single nucleotide polymorphism (SNP) of LUSC on CRC (**D**), CC (**E**) and RC (**F**) risk. **G**, **H**, **I** Plots of the effect size of each single nucleotide polymorphism (SNP) of LUAD on CRC (**G**), CC (**H**) and RC (**I**) risk. *LC* lung cancer, *CRC* colorectal cancer, *IVW* inverse variance weighted, *LUAD* adenocarcinoma of lung, *LUSC* squamous cell lung carcinoma, *CC* colon cancer, *RC* rectal cancer

In forward-direction MR, in overall LC to overall CRC MR study, IVW analysis revealed a significant association between overall LC and overall CRC at the genetic level (IVW: OR = 1.0026; 95% CI 1.0009–1.0043, *P* = 0.0029; Figs. 2A, 3A). The simple median method, weighted median approach, MR-PRESSO approach and MR-RAPS method all showed significant evidence of an association between overall LC and overall CRC (simple median: OR = 1.0035, 95% CI 1.0014–1.0057, *P* = 0.0012; weighted median: OR = 1.0040, 95% CI 1.0020–1.0060, *P* = 0.0001; MR-PRESSO: OR = 1.0026, 95% CI 1.0009–1.0043, *P* = 0.0080; MR-RAPS: OR = 1.0026, 95% CI 1.0013–1.0040, *P* = 0.0002; Fig. 2A).

In overall LC to colon cancer MR study, we have also identified a significant genetic association between overall LC and colon cancer (IVW: OR = 1.0014, 95% CI 1.0005–1.0023, *P* = 0.0028; simple median: OR = 1.0016, 95% CI 1.0003–1.0030, *P* = 0.014; weighted median: OR = 1.0016, 95% CI 1.00003–1.0029, *P* = 0.0160; MR-PRESSO: OR = 1.0014, 95% CI 1.0005–1.0023, *P* = 0.0054; MR-RAPS: OR = 1.0014, 95% CI 1.0005–1.0024, *P* = 0.0030; Figs. 2B, 3B).

In overall LC to rectal cancer MR study, we obtained consistent findings that the genetic level of overall LC was significantly correlated with rectal cancer (IVW: OR = 1.5979, 95% CI 1.1409–2.2380, *P* = 0.0064; simple median: OR = 1.5187, 95% CI 1.0667–2.1622, *P* = 0.0204; weighted median: OR = 1.5023, 95% CI 1.0718–2.1058, *P* = 0.0182; MR-PRESSO: OR = 1.5979; 95% CI 1.1409–2.2380, *P* = 0.0134; MR-RAPS: OR = 1.6269, 95% CI 1.2850–2.0597, *P* = 0.0001; Figs. 2C, 3C).

#### MR results of LUSC to CRC

In the study of LUSC and overall CRC MR, we have discovered a significant genetic correlation between LUSC and overall CRC (IVW: OR = 1.0017, 95% CI 1.0006–1.0028, *P* = 0.0022; simple median: OR = 1.0025, 95% CI 1.0011–1.0039, *P* = 0.0007; weighted median: OR = 1.0025, 95% CI 1.0011–1.0039, *P* = 0.0005; MR-PRESSO: OR = 1.0017, 95% CI 1.0006–1.0028, *P* = 0.0084; MR-RAPS: OR = 1.0017, 95% CI 1.0008–1.0027, *P* = 0.0002; Figs 2D, 3D).

In LUSC to colon cancer MR study, we have identified a significant genetic correlation between the two diseases (IVW: OR = 1.0008, 95% CI 1.0002–1.0014, *P* = 0.0133; simple median: OR = 1.0011, 95% CI 1.0002–1.0021, *P* = 0.0172; weighted median: OR = 1.0010, 95% CI 1.0001–1.0019, *P* = 0.0304; MR-PRESSO: OR = 1.0008, 95% CI 1.0002–1.0014, *P* = 0.0266; MR-RAPS: OR = 1.0008, 95% CI 1.0002–1.0014, *P* = 0.0138; Figs. 2E, 3E).

In LUSC to rectal cancer MR study, we have discovered a significant genetic correlation between these two cancers (IVW: OR = 1.3719; 95% CI 1.0687–1.7613; *P* = 0.0131; simple median: OR = 1.3616; 95% CI 1.0249–1.8089; *P* = 0.0332; weighted median: OR = 1.2885; 95% CI 1.0089–1.6456; *P* = 0.0422; MR-PRESSO: OR = 1.3719; 95% CI 1.0687–1.7613; *P* = 0.0264; MR-RAPS: OR = 1.3871; 95% CI 1.1745–1.6383; *P* = 0.0001; Figs 2F, 3F).

#### MR results of LUAD to CRC

In our investigation of LUAD and overall CRC MR, we did not observe any significant genetic association between LUAD and overall CRC (IVW: OR = 0.9993; 95% CI 0.9975–1.0010; *P* = 0.4092; simple median: OR = 1.00001; 95% CI 0.9979–1.0021; *P* = 0.9956; weighted median: OR = 0.9990; 95% CI 0.9970–1.0011; *P* = 0.3512; MR-PRESSO: OR = 0.9993; 95% CI 0.9975–1.0010; *P* = 0.4306; MR-RAPS: OR = 0.9993; 95% CI 0.9978–1.0007; *P* = 0.2932; Figs. 2G, 3G).

In our MR study of LUAD to colon cancer, we did not observe a significant genetic correlation between the two diseases (IVW: OR = 1.0004; 95% CI 0.9993–1.0014; *P* = 0.5073; simple median: OR = 1.0007; 95% CI 0.9992–1.0021; *P* = 0.3669; weighted median: OR = 1.0003; 95% CI 0.9990–1.0016; *P* = 0.6627; MR-PRESSO: OR = 1.0004; 95% CI = 0.9993–1.0014; *P* = 0.5239; MR-RAPS: OR = 1.0004; 95% CI 0.9994–1.0014; *P* = 0.4726; Figs. 2H, 3H).

In LUAD to rectal cancer MR study, we have not discovered a significant correlation between LUAD and rectal cancer at genetic level (IVW: OR = 0.8882; 95% CI 0.7156–1.1026; *P* = 0.2825; simple median: OR = 0.8301; 95% CI 0.6190–1.1132; *P* = 0.2136; weighted median: OR = 0.8268; 95% CI 0.6180–1.1063; *P* = 0.2005; MR-PRESSO: OR = 0.8882; 95% CI 0.7576–1.0414; *P* = 0.1783; MR-RAPS: OR = 0.8874; 95% CI 0.7114–1.1068; *P* = 0.2892; Fig. 2I, 3I).

### MR analysis results of CRC to LC

#### Screen and validation of IVs

In CRC to LC MR analysis, 56 overall CRC, 45 colon cancer and 29 rectal cancer IVs in the GWAS study reached significant differences (5 × 10^–8^). A single palindromic sequence has been identified within the SNPs datasets (overall CRC, colon cancer and rectal cancer: rs11874392). Based on the LD status between genetic variant loci, 50, 39, and 25 independent IVs associated with overall CRC, colon cancer and rectal cancer were selected without LD correlation (5 overall CRC, 5 colon cancer and 3 rectal cancer IVs are not LD independent. r^2^ < 0.01, window = 500 kb). Removal of IVs associated with confounders using the PhenoScanner database (smoking: rs597808; alcohol consumption: rs174533; BMI: rs1446585, rs597808, rs174533, rs1446585). Ultimately, we identified 47 genetic IVs for overall CRC, 37 for colon cancer and 25 for rectal cancer (Additional file 3: Table S2).

#### MR results of overall CRC to LC

**Fig. 4:**
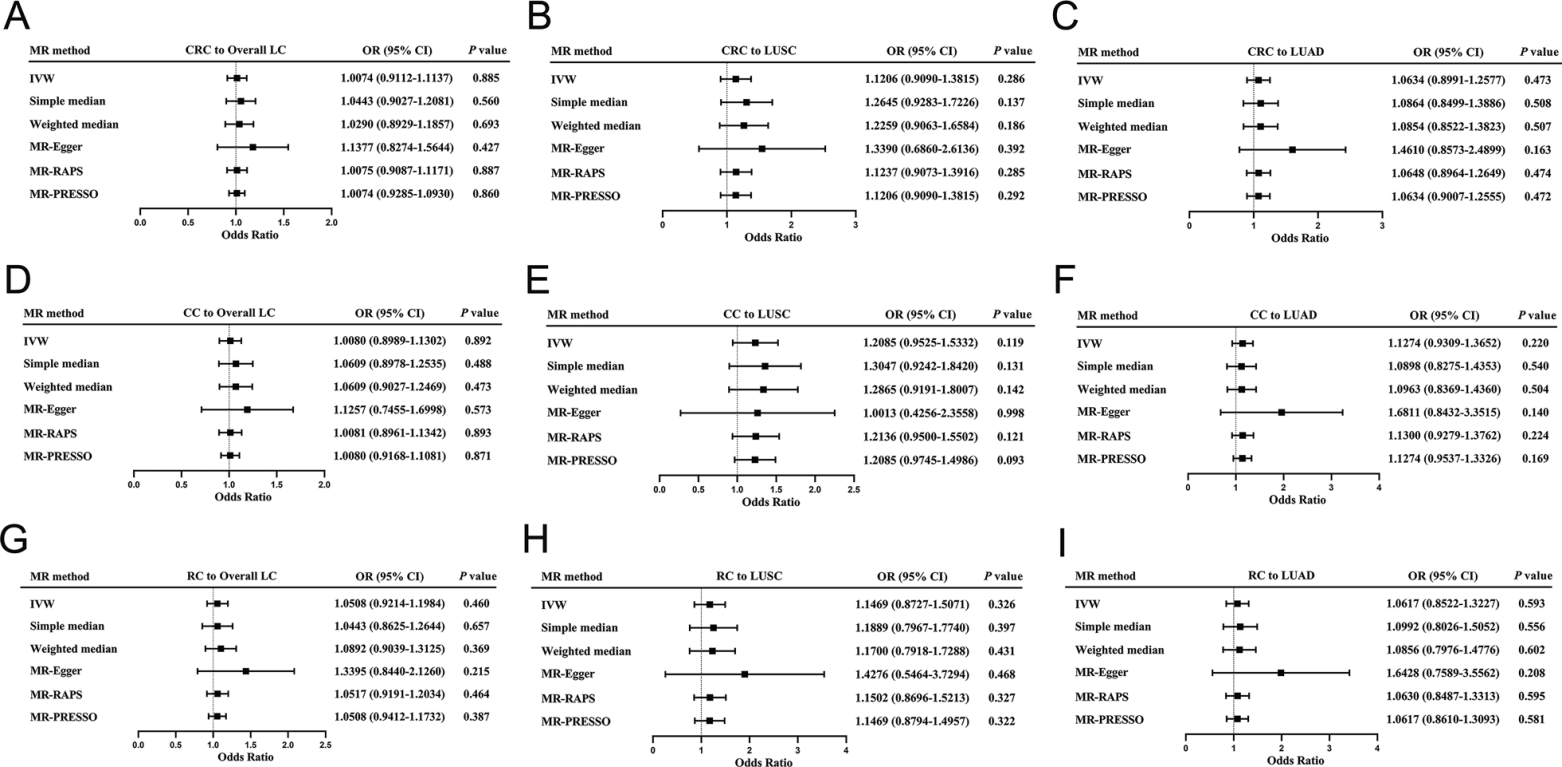
Forest plot of Two-Sample Mendelian Randomization study based on the MR method form CRC to LC. **A**, **B**, **C** Mendelian randomization estimates of genetically predicted CRC on overall LC (**A**), LUSC (**B**) and LUAD (**C**) risk. **D**, **E**, **F** Mendelian randomization estimates of genetically predicted CC on overall LC (**D**), LUSC (**E**) and LUAD (**F**) risk. **G**, **H**, **I** Mendelian randomization estimates of genetically predicted RC on overall LC (**G**), LUSC (**H**) and LUAD (**I**) risk. *LC* lung cancer, *CRC* colorectal cancer, *IVW* inverse variance weighted, *MR-PRESSO* Mendelian Randomization Pleiotropy RESidual Sum and Outlier, *MR-RAPS* Mendelian Randomization robust adjusted profile score, *LUAD* adenocarcinoma of lung, *LUSC* squamous cell lung carcinoma, *CC* colon cancer, *RC* rectal cancer

**Fig. 5:**
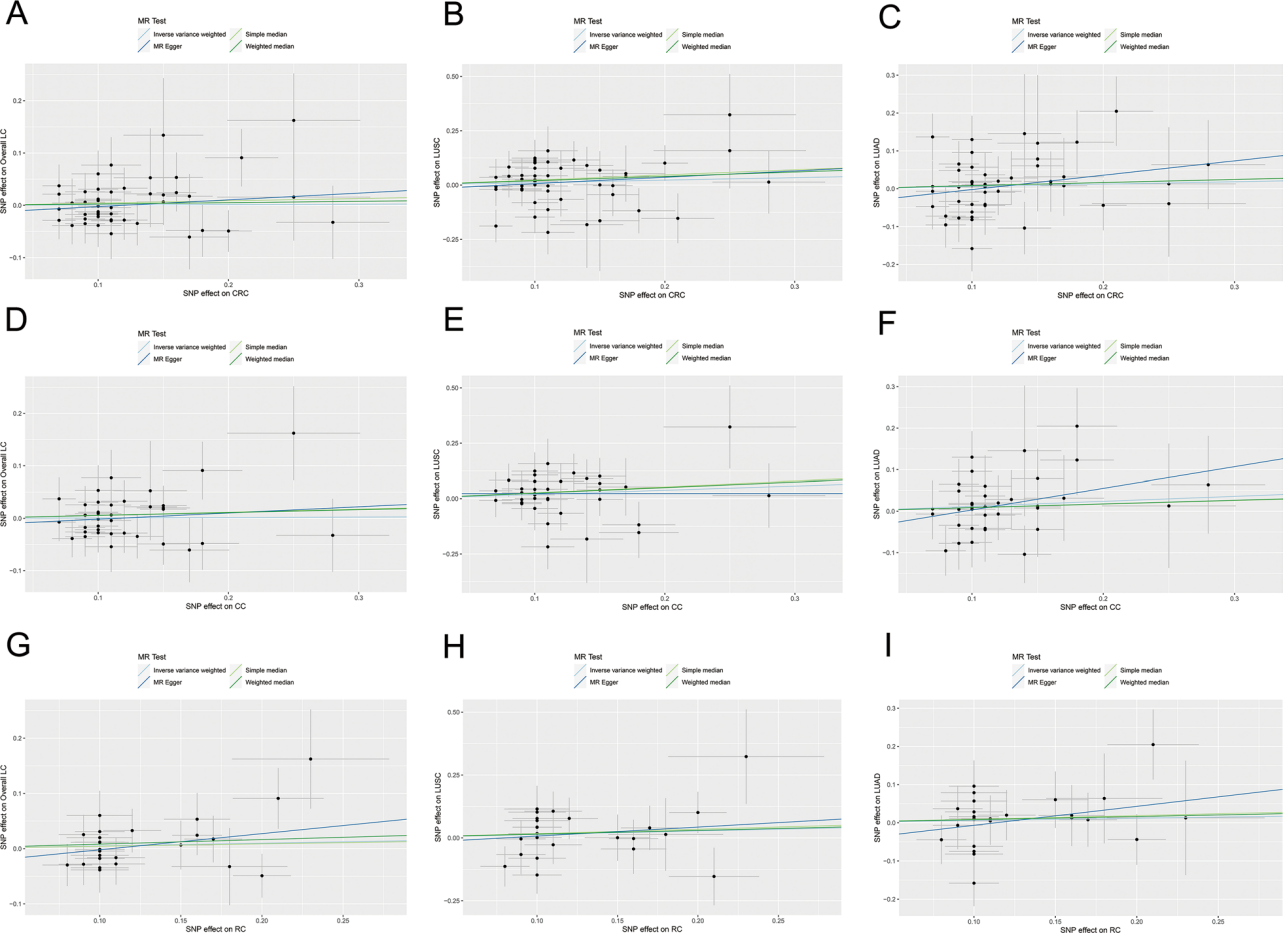
The scatterplots represent genetic IVs association between CRC and LC (Reverse MR analysis). **A**, **B**, **C** Plots of the effect size of each single nucleotide polymorphism (SNP) of CRC on overall LC (**A**), LUSC (**B**) and LUAD (**C**) risk. **D**, **E**, **F** Plots of the effect size of each single nucleotide polymorphism (SNP) of CC on overall LC (**D**), LUSC (**E**) and LUAD (**F**) risk. **G**, **H**, **I** Plots of the effect size of each single nucleotide polymorphism (SNP) of RC on overall LC (**G**), LUSC (**H**) and LUAD (**I**) risk. *LC* lung cancer, *CRC* colorectal cancer, *IVW* inverse variance weighted, *LUAD* adenocarcinoma of lung, *LUSC* squamous cell lung carcinoma, *CC* colon cancer, *RC* rectal cancer

About the reverse-direction MR, in overall CRC to overall LC MR study, we did not observe any significant genetic association between overall CRC and overall LC (IVW: OR = 1.0074; 95% CI 0.9112–1.1137; *P* = 0.8852; simple median: OR = 1.0443; 95% CI 0.9027–1.2081; *P* = 0.5599; weighted median: OR = 1.029; 95% CI 0.8929–1.1857; *P* = 0.6930; MR-PRESSO: OR = 1.0074; 95% CI 0.9285–1.0930; *P* = 0.8598; MR-RAPS: OR = 1.0075; 95% CI 0.9870–1.1171; *P* = 0.8866; Figs. 4A, 5A).

In our MR study of overall CRC to LUSC, we did not observe a significant genetic correlation between the two diseases (IVW: OR = 1.1206; 95% CI 0.909–1.3815; *P* = 0.2861; simple median: OR = 1.2645; 95% CI 0.9283–1.7226; *P* = 0.1367; weighted median: OR = 1.2259; 95% CI 0.9063–1.6584; *P* = 0.1864; MR-PRESSO: OR = 1.1206; 95% CI 0.909–1.3815; *P* = 0.2917; MR-RAPS: OR = 1.1237; 95% CI 0.9073–1.3916; *P* = 0.2852; Figs. 4B, 5B).

In overall CRC to LUAD MR study, we have not discovered a significant genetic correlation between these two diseases (IVW: OR = 1.0634; 95% CI 0.8991–1.2577; *P* = 0.4730; simple median: OR = 1.0864; 95% CI 0.8499–1.3886; *P* = 0.5082; weighted median: OR = 1.0854; 95% CI 0.8522–1.3823; *P* = 0.5068; MR-PRESSO: OR = 1.0634; 95% CI 0.9007–1.2555; *P* = 0.4719; MR-RAPS: OR = 1.0648; 95% CI 0.8964–1.2649; *P* = 0.4745; Figs. 4C, 5C).

#### MR results of colon cancer to LC

In colon cancer and overall LC MR study, we did not obtain any statistically significant association between colon cancer and overall LC at genetic level (IVW: OR = 1.008; 95% CI 0.8989–1.1302; *P* = 0.8921; simple median: OR = 1.0609; 95% CI 0.8978–1.2535; *P* = 0.4876; weighted median: OR = 1.0609; 95% CI 0.9027–0.9027; *P* = 0.4727; MR-PRESSO: OR = 1.008; 95% CI 0.9168–1.1081; *P* = 0.8708; MR-RAPS: OR = 1.0081; 95% CI 0.8961–1.1342; *P* = 0.8931; Figs. 4D, 5D).

In colon cancer to LUSC, we did not detect a significant genetic correlation between the two cancers (IVW: OR = 1.1206; 95% CI 0.909–1.3815; *P* = 0.2861; simple median: OR = 1.2645; 95% CI 0.9283–1.7226; *P* = 0.1367; weighted median: OR = 1.2259; 95% CI 0.9063–1.6584; *P* = 0.1864; MR-PRESSO: OR = 1.1206; 95% CI 0.909–1.3815; *P* = 0.2917; MR-RAPS: OR = 1.1237; 95% CI 0.9073–1.3916; *P* = 0.2852; Figs. 4E, 5E).

In colon cancer to LUAD MR study, we have also not found a significant correlation between these two diseases at genetic level (IVW: OR = 1.1274; 95% CI 0.9309–1.3652; *P* = 0.2197; simple median: OR = 1.0898; 95% CI 0.8275–1.4353; *P* = 0.5404; weighted median: OR = 1.0963; 95% CI 0.8369–1.436; *P* = 0.5045; MR-PRESSO: OR = 1.1274; 95% CI 0.9537–1.3326; *P* = 0.1687; MR-RAPS: OR = 1.13; 95% CI 0.9279–1.3762; *P* = 0.2240; Figs. 4F, 5F).

#### MR results of rectal cancer to LC

In rectal cancer and overall LC MR, we did not found significant genetic association between rectal cancer and overall LC (IVW: OR = 1.0508; 95% CI 0.9214–1.1984; *P* = 0.4596; simple median: OR = 1.0443; 95% CI 0.8625–1.2644; *P* = 0.6570; weighted median: OR = 1.0892; 95% CI 0.9039–1.3125; *P* = 0.3692; MR-PRESSO: OR = 1.0508; 95% CI 0.9412–1.1732; *P* = 0.3867; MR-RAPS: OR = 1.0517; 95% CI 0.9191–1.2034; *P* = 0.4636; Figs. 4G, 5G).

In rectal cancer to LUSC MR, we did not obtain a significant correlation between the two diseases at genetic level (IVW: OR = 1.1469; 95% CI 0.8727–1.5071; *P* = 0.3255; simple median: OR = 1.1889; 95% CI 0.7967–1.774; *P* = 0.3969; weighted median: OR = 1.17; 95% CI 0.7918–1.7288; *P* = 0.4307; MR-PRESSO: OR = 1.1469; 95% CI 0.8794–1.4957; *P* = 0.3219; MR-RAPS: OR = 1.1502; 95% CI 0.8696–1.5213; *P* = 0.3267; Figs. 4H, 5H).

In rectal cancer to LUAD MR study, we have not discovered a significant genetic correlation between rectal cancer and LUAD (IVW: OR = 1.0617; 95% CI 0.8522–1.3227; *P* = 0.5933; simple median: OR = 1.0992; 95% CI 0.8026–1.5052; *P* = 0.5556; weighted median: OR = 1.0856; 95% CI 0.7976–1.4776; *P* = 0.6016; MR-PRESSO: OR = 1.0617; 95% CI 0.861–1.3093; *P* = 0.5806; MR-RAPS: OR = 1.063; 95% CI 0.8487–1.3313; *P* = 0.5949; Figs 4I, 5I).

### Horizontal pleiotropy and heterogeneity test

In LC overall and LUSC to rectal cancer MR analysis, Cochrane’s Q tests showed that there was some heterogeneity between the LC overall and LUSC IVs (LC overall: Q = 40.737, *P* = 0.003; LUSC: Q = 32.833, *P* = 0.003; Additional file 2: Table S1). The leave-one-out plot indicated that no single SNP drove the genetic association in LC overall and LUSC to rectal cancer MR (Additional file 1: Fig. S1). No heterogeneity was found in any other MR analysis group.

The MR-Egger regression analysis showed that the horizontal pleiotropy of the IVs was present in LUAD to CRC overall and colon cancer MR analysis (CRC overall: *P* = 0.019; colon cancer: *P* = 0.048; Additional file 2: Table S1). No IVs with horizontal pleiotropy were found by MR-PRESSO method in LUAD to CRC overall and colon cancer MR analysis. No horizontal pleiotropy was found in any other MR analysis group.

### GRS analysis results

#### GRS_LC_ to CRC

Consistent with the MR results of LC to CRC, GRS_overall LC_ shows association between overall LC and CRC (overall CRC, colon cancer and rectal cancer) at the genetic level (overall CRC: OR = 1.0026, 95% CI 1.0012–1.0039, *P* = 0.0002; colon cancer: OR = 1.0014, 95% CI 1.0005–1.0023, *P* = 0.0028; rectal cancer: OR = 1.5979, 95% CI 1.2695–2.0013, *P* = 6.53E-05) (Table 1). Similarly, GRS_LUSC_ shows association between LUSC and CRC (overall CRC, colon cancer and rectal cancer) at the genetic level (overall CRC: OR = 1.0017, 95% CI 1.0008–1.0026, *P* = 0.0002; colon cancer: OR = 1.0008, 95% CI 1.0002–1.0014, *P* = 0.01; rectal cancer: OR = 1.3719, 95% CI 1.1654–1.6150, *P* = 0.0001) (Table 1). However, GRS_LUAD_ does not found any correlation between LUAD and CRC (overall CRC, colon cancer and rectal cancer) at the genetic level (overall CRC: OR = 0.9993, 95% CI 0.9979–1.0007, *P* = 0.3; colon cancer: OR = 1.0004, 95% CI 0.9994–1.0013, *P* = 0.47; rectal cancer: OR = 0.8882, 95% CI 0.7156–1.1026, *P* = 0.28) (Table 1).

**Table 1:** The effects of the GRS_LC_ on CRC and the GRS_CRC_ on LC

Exposure	Outcome	OR (95% CI)	*P* value	Exposure	Outcome	OR (95% CI)	*P* value
Overall LC	Overall CRC	1.0026 (1.0012–1.0039)	0.0002	Overall CRC	Overall LC	1.0074 (0.9112–1.1137)	0.89
	Colon cancer	1.0014 (1.0005–1.0023)	0.0028		LUSC	1.1206 (0.9096–1.3806)	0.28
	Rectal cancer	1.5979 (1.2695–2.0113)	6.53E-05		LUAD	1.0634 (0.8991–1.2577)	0.47
LUSC	Overall CRC	1.0017 (1.0008–1.0026)	0.0002	Colon cancer	Overall LC	1.0080 (0.8989–1.1302)	0.89
	Colon cancer	1.0008 (1.0002–1.0014)	0.01		LUSC	1.2085 (0.9525–1.5332)	0.12
	Rectal cancer	1.3719 (1.1654–1.6150)	0.0001		LUAD	1.1274 (0.9309–1.3652)	0.22
LUAD	Overall CRC	0.9993 (0.9979–1.0007)	0.3	Rectal cancer	Overall LC	1.0508 (0.9214–1.1984)	0.46
	Colon cancer	1.0004 (0.9994–1.0013)	0.47		LUSC	1.1469 (0.8727–1.5072)	0.33
	Rectal cancer	0.8882 (0.7156–1.1026)	0.28		LUAD	1.0617 (0.8522–1.3227)	0.59

#### GRS_CRC_ to LC

For the GRS_CRC_ to LC analysis, the results showed that no association between overall CRC and LC (overall LC, LUSC and LUAD) at the genetic level (overall LC: OR = 1.0074, 95% CI 0.9112–1.1137, *P* = 0.89; LUSC: OR = 1.1206, 95% CI 0.9096–1.3806, *P* = 0.28; LUAD: OR = 1.0634, 95% CI 0.8991–1.2577, *P* = 0.47) (Table 1). Same as above, GRS_colon cancer_ shows no association between colon cancer and LC (overall LC, LUSC and LUAD) at the genetic level (overall LC: OR = 1.0080, 95% CI 0.8989–1.1302, *P* = 0.89; LUSC: OR = 1.2085, 95% CI 0.9525–1.5332, *P* = 0.12; LUAD: OR = 1.1274, 95% CI 0.9309–1.3652, *P* = 0.22) (Table 1). Similarly, GRS_rectal cancer_ does not discover any correlation between rectal cancer and LC (overall LC, LUSC and LUAD) at the genetic level (overall LC: OR = 1.0508, 95% CI 0.9214–1.1984, *P* = 0.46; LUSC: OR = 1.1469, 95% CI 0.8727–1.5072, *P* = 0.33; LUAD: OR = 1.0617, 95% CI 0.8522–1.3227, *P* = 0.59) (Table 1). The result of GRS_CRC_ to LC was consistent with the above MR results of CRC to LC.

## Discussion

SPC refers to the occurrence of a new primary cancer in an individual previously diagnosed with and treated for another cancer. In recent years, advancements in cancer prevention, diagnosis, and treatment have significantly increased early-stage cancer patients receiving prompt and effective care. As a result, there has been a notable improvement in long-term survival rates, with 14.5 million individuals surviving early-stage cancers alone in the United States in 2014 [24]. Previous research has demonstrated that the incidence of SPC is significantly higher in cancer patients than in the general population and tends to increase with longer survival times. After 20 years or more of follow-up, over 19% of patients are likely to develop SPC [33]. Regarding PLC, early-stage patients have a 1.7-fold higher risk of developing SPC than the general population, and approximately 13.4–22% of patients will develop SPC [34, 35]. As the incidence of SPC following early LC surgery is progressively increasing, researchers have shown significant interest in studying the morbidity, treatment, and prognosis of SPCs. Given that CRC has the highest morbidity and mortality rate among SPCs, investigating the association between PLC and CRC can aid in identifying high-risk patients for early screening after LC surgery and providing timely and effective treatment, ultimately improving patient survival rates.

The etiology of SPC remains uncertain, and observational studies indicate a potential association between genetic predisposition, environmental influences, and lifestyle choices in the development of SPC. Previous observational studies have suggested a possible association between PLC and CRC [36]. However, due to the presence of various confounding factors and the challenges associated with conducting large-scale case–control and cohort studies, the clinical question of whether there is indeed an association between PLC and CRC and its extent remains to be explored. A study by Zhou et al.[37], based on the SEER database, reported that patients with LC had a 19% higher risk of developing CRC than the general population, and patients with LUSC had a 38% higher risk of CRC than the general population. However, there was no difference in the risk of CRC between patients with LUAD and the general population. However, Su et al.’s retrospective study found no increased risk of CRC among survivors of PLC [38]. Meanwhile, in 2009, Noura et al. surveyed 301 patients with CRC to assess post-operative SPC (extra-CRC) occurrence. The results showed that the incidence of postoperative extra-CRC in CRC patients was significantly higher than that in normal population, especially LC. During the 10 year follow-up period, a total of 40 cases of secondary primary extra-CRC (including LC, stomach cancer, liver cancer, etc.) occurred, of which 8 cases (20%) were LC, ranking first [39]. The present study is an innovative approach to exploring the association between PLC and CRC using a two-sample MR study.

In our study we have identified a significant association between CRC and the occurrence of overall LC and LUSC for the first time through stratified analysis of PLC by two-sample MR approach. We found an increased risk of SPC-CRC following PLC, especially LUSC. To investigate the underlying reasons, a PLC GWAS conducted by James et al. in 2017 demonstrated significant genomic differences between LUAD and LUSC, despite both belonging to NSCLC, suggesting potential distinct mechanisms for the development of LUAD and LUSC [13]. Furthermore, multiple previous studies have indicated the presence of shared signaling pathways, such as the PI3K pathway [40, 41], FGFR1 pathway [42, 43] between LUSC and CRC, implying potential common genetic origins and developmental processes between these two cancer types.

The 2021 United States Preventive Services Task Force (USPSTF) [44] recommends that all adults aged 50 to 75 undergo CRC screening. For individuals with a family history of CRC, the population with obesity, long-term smoking, and heavy alcohol consumption, regular screening is recommended due to the higher risk of developing CRC. Additionally, even in the absence of these risk factors, the USPSTF recommends starting CRC screening at age 45, with options including annual high-sensitivity guaiac-based fecal occult blood test (gFOBT) or fecal immunochemical test (FIT), every 1 to 3 years stool DNA-FIT testing, every 5 years computed tomography colonography, every 5 years flexible sigmoidoscopy, every 10 years colonoscopy, and annual FIT. Our research conclusions validate the results of previous observational studies [37]. Therefore, for individuals with a history of PLC, regular screening should be conducted, including fecal occult blood test, digital rectal examination, and colonoscopy. Close attention should be paid to the occurrence of SPC-CRC in order to initiate early intervention and treatment.

There are several advantages in our MR study. Firstly, to the best of our knowledge, this is the first study to evaluate the genetic association between LC and CRC based on a two sample MR analysis with large scale GWAS data. Compared to previous observational studies, MR analysis could effectively reduce potential bias including confounders and reverse causation, thus enhancing the causal inference. Secondly, GWAS datasets of LC and CRC applied were predominately based on populations of European ancestry, which was capable to minimize the impact of population stratification. Furthermore, we systematically screened confounding factors associated with PLC and CRC using the PhenoScanner database and eliminated IVs associated with confounding factors to avoid the potential horizontal pleiotropy of genetic IVs. Meanwhile, MR-Egger and MR-PRESSO (Outlier-corrected) outlier SNP evaluation methods were used to examine the influence of pleiotropy further and ensure the reliability of the results [45, 46]. In addition, Cochran's Q and leave-one-out method was employed to examine heterogeneity in IVs. If Cochran's Q test detected no significant heterogeneity, an IVW linear regression was utilized for unbiased association estimation; if significant heterogeneity existed, a random-effects IVW model was applied to ensure the accuracy of results [22, 47]. Finally, besides employing the IVW method as the primary analysis approach, we also utilized the GRS method as a secondary analysis in this study. Moreover, various MR complementary methods were employed to ensure result accuracy, including the weight median, simple median, MR-RAPS, and MR-PRESSO methods. However, we would like to acknowledge some limitations. Firstly, the study included a single population, and the representativeness of the results remains to be further verified in the whole population. Secondly, although a series of strict steps were used to identify outlier variants for avoiding horizontal pleiotropy, we still unable to totally eliminate the impact of horizontal pleiotropy, which may be due to the complex and unclear biological function of many genetic variants. Thirdly, as we explore the relationship between LC and rectal cancer, we achieved a statistical efficacy of more than 80%, whereas in our study of LC and colon cancer, it was less than 80%. And larger sample sizes and more advanced methods are needed to corroborate the results and fully illustrate the statistical power. Finally, GWAS could provide new insights into genes involved in PLC-CRC, but the precise mechanisms studies are needed for better understanding the pathophysiology.

In summary, this study has established a genetic association between PLC and CRC, which provides an essential basis for the precise prevention of SPC-CRC after PLC, suggesting that we should pay more attention to the incidence of SPC-CRC and carry out intervention and treatment as soon as possible.

### Supplementary Information

**Additional file 1: Figure S1.** MR leave-one-out sensitivity analysis for “exposure” on “outcome”.

**Additional file 2: Table S1.** Results of heterogeneity test, horizontal pleiotropy test. LC: lung cancer; CRC: colorectal cancer; LUAD: adenocarcinoma of lung; LUSC: squamous cell lung carcinoma.

**Additional file 3: Table S2.** Effect estimates for associations of genetic instruments with exposure.

## Data Availability

The original contributions presented in the study are included in the article/Additional file, further inquiries can be directed to the corresponding author.

## Abbreviations

LC: Lung cancer
LUSC: Squamous cell lung carcinoma
LUAD: Adenocarcinoma of the lung
CRC: Colorectal cancer
SPC: Second primary cancer
PLC: Primary lung cancer
IVs: Instrumental variables
GWAS: Genome-wide association studies
MR: Mendelian randomization
IVW: Inverse-variance weighted
GRS: Genetic risk score
MR-RAPS: Mendelian randomization robust adjusted profile score
MR-PRESSO: Mendelian randomization pleiotropy residual sum and outlier
SNP: Single nucleotide polymorphism
GWAS: Genome-wide association study
InSIDE: Instrument strength independent of direct effect

## References

[1] Sung H (2021). Global cancer statistics 2020: GLOBOCAN estimates of incidence and mortality worldwide for 36 cancers in 185 countries. CA Cancer J Clin.

[2] Luo L (2022). Risk factors and prognostic nomogram for patients with second primary cancers after lung cancer using classical statistics and machine learning. Clin Exp Med.

[3] Jonas DE (2021). Screening for lung cancer with low-dose computed tomography: updated evidence report and systematic review for the us preventive services task force. JAMA.

[4] Okami J (2019). Demographics, safety and quality, and prognostic information in both the seventh and eighth editions of the TNM classification in 18,973 surgical cases of the Japanese joint committee of lung cancer registry database in 2010. J Thorac Oncol.

[5] Vansteenkiste J (2014). 2nd ESMO consensus conference on lung cancer: early-stage non-small-cell lung cancer consensus on diagnosis, treatment and follow-up. Ann Oncol.

[6] Goldstraw P (2016). The IASLC lung cancer staging project: proposals for revision of the TNM stage groupings in the forthcoming (Eighth) edition of the TNM classification for lung cancer. J Thorac Oncol.

[7] Saji H (2022). Segmentectomy versus lobectomy in small-sized peripheral non-small-cell lung cancer (JCOG0802/WJOG4607L): a multicentre, open-label, phase 3, randomised, controlled, non-inferiority trial. Lancet.

[8] Rice D (2003). The risk of second primary tumors after resection of stage I nonsmall cell lung cancer. Ann Thorac Surg.

[9] Hariton E (2018). Randomised controlled trials - the gold standard for effectiveness research: Study design: randomised controlled trials. BJOG.

[10] Davey Smith G (2014). Mendelian randomization: genetic anchors for causal inference in epidemiological studies. Hum Mol Genet.

[11] Li C (2021). Rheumatoid arthritis decreases risk for Parkinson’s disease: a Mendelian randomization study. NPJ Parkinsons Dis.

[12] Zhu T (2021). Polycystic ovary syndrome and breast cancer subtypes: a Mendelian randomization study. Am J Obstet Gynecol.

[13] McKay JD (2017). Large-scale association analysis identifies new lung cancer susceptibility loci and heterogeneity in genetic susceptibility across histological subtypes. Nat Genet.

[14] Huyghe JR (2019). Discovery of common and rare genetic risk variants for colorectal cancer. Nat Genet.

[15] Didelez V (2007). Mendelian randomization as an instrumental variable approach to causal inference. Stat Methods Med Res.

[16] Zhou W (2021). Causal relationships between body mass index, smoking and lung cancer: univariable and multivariable Mendelian randomization. Int J Cancer.

[17] Chen L (2022). Insights into modifiable risk factors of cholelithiasis: a Mendelian randomization study. Hepatology.

[18] Kamat MA (2019). PhenoScanner V2: an expanded tool for searching human genotype-phenotype associations. Bioinformatics.

[19] Burgess S (2015). Using published data in Mendelian randomization: a blueprint for efficient identification of causal risk factors. Eur J Epidemiol.

[20] Burgess S (2013). Mendelian randomization analysis with multiple genetic variants using summarized data. Genet Epidemiol.

[21] Thomas DC (2004). Commentary: the concept of 'Mendelian randomization'. Int J Epidemiol.

[22] Bowden J (2017). A framework for the investigation of pleiotropy in two-sample summary data Mendelian randomization. Stat Med.

[23] Xiao Z (2023). Investigation of the causal relationship between alcohol consumption and COVID-19: a two-sample Mendelian randomization study. Int J Comput Intell Syst.

[24] Verbanck M (2018). Detection of widespread horizontal pleiotropy in causal relationships inferred from Mendelian randomization between complex traits and diseases. Nat Genet.

[25] Liu Y (2022). Smoking, alcohol consumption, diabetes, body mass index, and peptic ulcer risk: a two-sample Mendelian randomization study. Front Genet.

[26] Zhao Q (2018). Statistical inference in two-sample summary-data Mendelian randomization using robust adjusted profile score. Ann Stat.

[27] Burgess S (2017). Interpreting findings from Mendelian randomization using the MR-egger method. Eur J Epidemiol.

[28] Luo Q (2020). Assessment causality in associations between serum uric acid and risk of schizophrenia: a two-sample bidirectional Mendelian randomization study. Clin Epidemiol.

[29] Palmer TM (2012). Using multiple genetic variants as instrumental variables for modifiable risk factors. Stat Methods Med Res.

[30] Hemani G (2018). The MR-base platform supports systematic causal inference across the human phenome. Elife.

[31] Fang Z (2022). The timing of adiposity and changes in the life course on the risk of cancer. Cancer Metastasis Rev.

[32] Caini S (2022). The prognostic impact of quitting smoking at or around diagnosis on the survival of patients with gastrointestinal cancers: a systematic literature review. Cancers.

[33] Zhong YJ (2019). Trends and patterns of disparities in burden of lung cancer in the United States, 1974–2015. Front Oncol.

[34] Vogt A (2017). Multiple primary tumours: challenges and approaches, a review. ESMO Open.

[35] Baum P (2022). Trends in age- and sex-specific lung cancer mortality in Europe and Northern America: analysis of vital registration data from the WHO mortality database between 2000 and 2017. Eur J Cancer.

[36] de Cos S, Escuín J (2016). Disease recurrence and second tumors in long-term survivors of lung cancer. Arch Bronconeumol.

[37] Zhou H (2019). Risk of second primary malignancy after non-small cell lung cancer: a competing risk nomogram based on the SEER database. Ann Transl Med.

[38] Su VY (2017). Risk of second primary malignancies in lung cancer survivors—the influence of different treatments. Target Oncol.

[39] Noura S (2009). Second primary cancer in patients with colorectal cancer after a curative resection. Dig Surg.

[40] Narayanankutty A (2019). PI3K/Akt/mTOR pathway as a therapeutic target for colorectal cancer: a review of preclinical and clinical evidence. Curr Drug Targets.

[41] Yue A (2023). Tastin promotes non-small-cell lung cancer progression through the ErbB4, PI3K/AKT, and ERK1/2 pathways. Exp Biol Med.

[42] Yin F (2022). Novel dual inhibitor for targeting PIM1 and FGFR1 kinases inhibits colorectal cancer growth in vitro and patient-derived xenografts in vivo. Acta Pharm Sin B.

[43] Weiss J (2010). Frequent and focal FGFR1 amplification associates with therapeutically tractable FGFR1 dependency in squamous cell lung cancer. Sci Transl Med.

[44] Davidson KW (2021). Screening for colorectal cancer: US preventive services task force recommendation statement. JAMA.

[45] Bowden J (2015). Mendelian randomization with invalid instruments: effect estimation and bias detection through egger regression. Int J Epidemiol.

[46] Burgess S (2017). Sensitivity analyses for robust causal inference from Mendelian randomization analyses with multiple genetic variants. Epidemiology.

[47] Wu F (2020). Mendelian randomization study of inflammatory bowel disease and bone mineral density. BMC Med.

