# Supplementary figures and images for: Bidirectional Mendelian randomization analysis of the genetic association between primary lung cancer and colorectal cancer
Source: J Transl Med. 2023 Oct 15;21:722. doi: 10.1186/s12967-023-04612-7 (PMC10577972; doi:10.1186/s12967-023-04612-7)

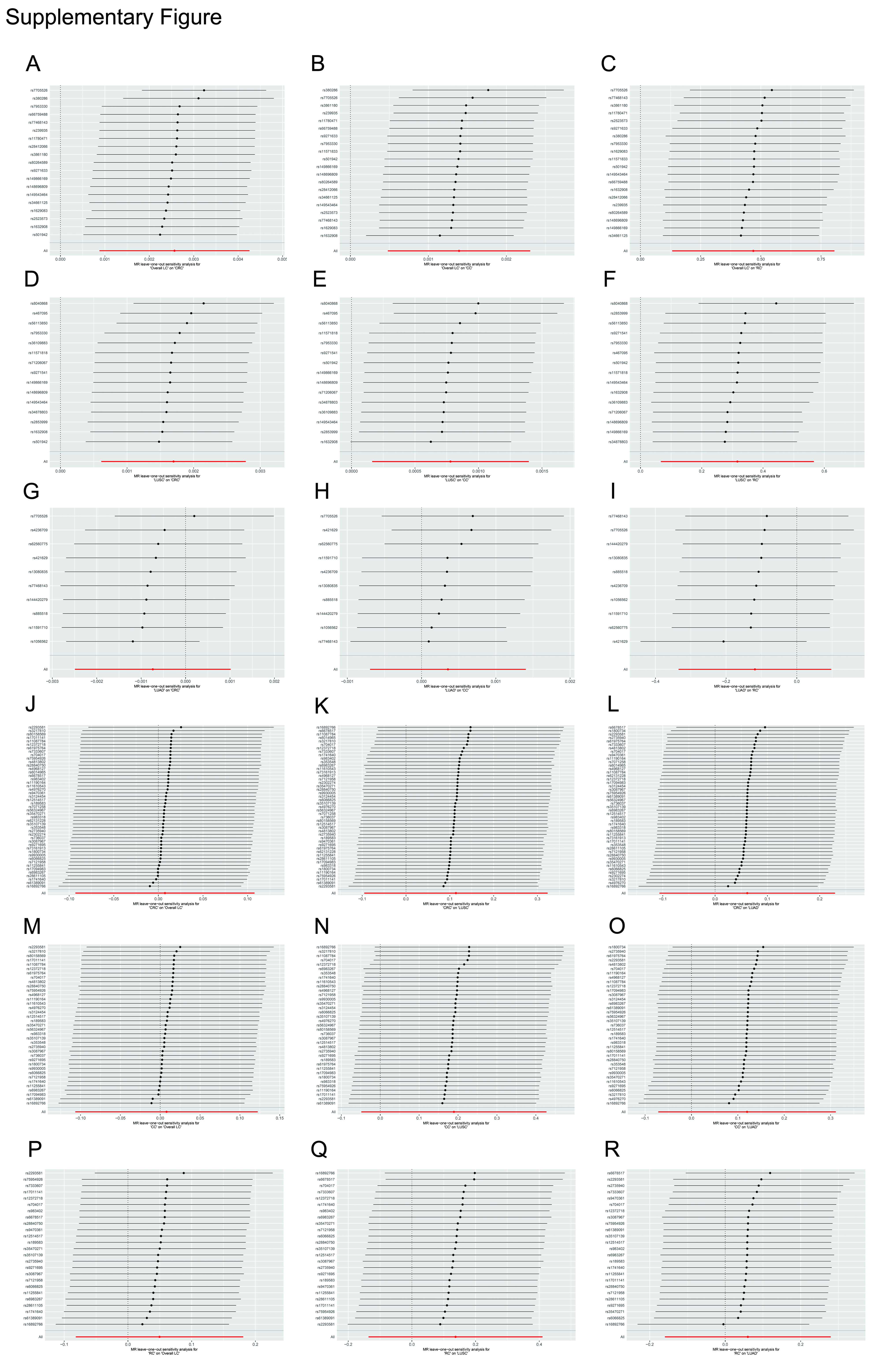

Supplement: Supplementary file 1 — Additional file 1: Figure S1. MR leave-one-out sensitivity analysis for “exposure” on “outcome”. [file 12967_2023_4612_MOESM1_ESM.tif]
