# Supplementary material for: Bidirectional Mendelian randomization analysis of the genetic association between primary lung cancer and colorectal cancer
Source: J Transl Med. 2023 Oct 15;21:722. doi: 10.1186/s12967-023-04612-7 (PMC10577972; doi:10.1186/s12967-023-04612-7)
Supplement: Supplementary file 2 — Additional file 2: Table S1. Results of heterogeneity test, horizontal pleiotropy test. LC: lung cancer; CRC: colorectal cancer; LUAD: adenocarcinoma of lung; LUSC: squamous cell lung carcinoma. [file 12967_2023_4612_MOESM2_ESM.docx]

| **Supplementary Table 1.** Results of heterogeneity test, horizontal pleiotropy test. | | | | | | | |
| --- | --- | --- | --- | --- | --- | --- | --- |
| **Exposure** | **Outcome** | **N_SNP_** | **MR-Egger**  **intercept *P*** | **Global Test** | **Global Test  *P*** | **Cochran’s Q** | **Cochran’s Q *P*** |
| LC Overall | CRC | 19 | 0.407 | 32.799 | 0.0628 | 28.275 | 0.058 |
| LUAD |  | 10 | 0.019 | 18.921 | 0.1182 | 14.311 | 0.112 |
| LUSC |  | 15 | 0.295 | 24.764 | 0.1054 | 20.587 | 0.113 |
| LC Overall | colon cancer | 20 | 0.847 | 19.762 | 0.5532 | 17.193 | 0.577 |
| LUAD |  | 10 | 0.048 | 13.423 | 0.309 | 10.531 | 0.309 |
| LUSC |  | 15 | 0.783 | 16.554 | 0.4424 | 13.988 | 0.451 |
| LC Overall | rectal cancer | 20 | 0.943 | 44.014 | 0.0032 | 40.737 | 0.003 |
| LUAD |  | 10 | 0.754 | 6.529 | 0.8156 | 4.875 | 0.845 |
| LUSC |  | 15 | 0.964 | 39.094 | 0.0052 | 32.833 | 0.003 |
| CRC | LC overall | 47 | 0.427 | 31.838 | 0.961 | 30.395 | 0.963 |
| colon cancer |  | 37 | 0.584 | 26.087 | 0.928 | 24.669 | 0.923 |
| rectal cancer |  | 25 | 0.283 | 18.624 | 0.838 | 16.883 | 0.854 |
| CRC | LUAD | 47 | 0.218 | 46.977 | 0.510 | 45.029 | 0.513 |
| colon cancer |  | 37 | 0.237 | 29.099 | 0.848 | 27.477 | 0.845 |
| rectal cancer |  | 25 | 0.248 | 23.761 | 0.604 | 21.823 | 0.590 |
| CRC | LUSC | 47 | 0.583 | 48.137 | 0.485 | 46.280 | 0.461 |
| colon cancer |  | 37 | 0.654 | 31.052 | 0.772 | 29.431 | 0.772 |
| rectal cancer |  | 25 | 0.641 | 24.490 | 0.554 | 22.677 | 0.539 |
